# Reflection of vortex beam from relativistic flying mirror

**DOI:** 10.1038/s41598-022-16450-6

**Published:** 2022-07-22

**Authors:** Weixin Chen, Xiaomei Zhang, Dirui Xu, Xinju Guo, Baifei Shen

**Affiliations:** Department of Physics, Shanghai Normal University, Shanghai, 200234 China

**Keywords:** Optics and photonics, Optical physics

## Abstract

In this study, the change in the angular momentum of a vortex beam after reflection from a relativistic flying mirror is investigated. This change is determined by performing full three-dimensional particle-in-cell simulations. The results confirm that the spin angular momentum and linear momentum carried by the reflected beam are collinear. In addition, we show that the orbital angular momentum is not collinear with the linear momentum carried by the reflected beam owing to the Doppler effect.

## Introduction

With the advancement of laser technology (e.g., chirped pulse amplification^1^), lasers with focal intensities above 10^22^ W/cm^2^ and durations shorter than 10 fs have been developed^2,3^. When an ultra-short and ultra-strong laser pulse impinges on a solid foil, the laser radiation pressure^4–6^ causes both the electrons in the target and the entire solid target, including electrons and protons, to move relativistically and produce an electron^7^ or plasma flying mirror^8–10^. The plasma flying mirror, or relativistic electron layer, reflects another incoming laser, thereby changing the laser parameters and yielding new physical phenomena, such as enhancing the laser pulse intensity and generating an ultra-short pulse^11–14^.

Light reflection at a planar interface is one of the most basic optical processes. Usually, light is reflected at the plane of incidence with an angle of reflection equal to its incident angle, following the well-known optical reflection law in the stationary frame of reference^15,16^. The speed of light is constant regardless of the motion of the observer or the light source, and it determines the relationships between the space and time coordinates in different inertial reference frames as Lorentz transformations^17^. However, the optical reflection law in the stationary frame of reference is not applicable when the reflecting plane moves relativistically. Because of the relativistic Doppler shift, which can be derived from the Lorentz transformation, the angle of reflection is not equal to the angle of incidence^17^. In 2009, Wu et al.^18^ studied the process of Gaussian beams obliquely impinging on a relativistic electron layer. They concluded that the frequency and emission angle of the reflected beam increased with increasing angle of incidence. With an increase in the velocity of the electron layer, the frequency increased, whereas the reflection angle decreased.

According to Maxwell's theory, both energy and momentum are carried by electromagnetic waves^17^. The momentum carried by photons includes both linear and angular contributions^19^. The angular momentum (AM) has a spin component associated with the polarization of light and an orbital component associated with spatial distribution. As only two polarization states exist, namely the right and left circular polarizations, the average spin angular momentum (SAM) carried by a photon is fundamentally limited to two values: $\hslash$ or $-\hslash$^20^, where $\hslash$ is the reduced Planck constant. As an intrinsic quantity, the direction of the SAM is expected to be parallel to the linear momentum (LM) of light^21^. In 1992, Allen et al.^22^ recognized that light beams with an azimuthal phase dependence of $\mathrm{exp}(il\varphi )$ can carry an orbital angular momentum (OAM), where $l$ is the azimuthal index and $\varphi$ is the azimuth angle. The average OAM carried by photon scaling with $l$ can be several times greater than the SAM. The direction of the OAM carried by the vortex beams is typically parallel to the direction of the LM. In the case of oblique incidence and a static reflecting plane, the direction of the AM changes after reflection because the AM is constant when it is under space inversion as a pseudovector^17^. The AM remains collinear with the LM carried by the reflected beam, but in the opposite direction.

In this study, inspired by the flying mirror and the generation of relativistic vortex beams^23–29^ with a large OAM, we re-examined the behavior of an intense vortex beam reflected by a relativistic flying mirror in the case of oblique incidence. In addition to the frequency and emission angle of the reflected beam, the OAM also changes. In this case, the OAM of the reflected beam is no longer collinear with LM, unlike the SAM. This physical phenomenon is expected to be used to generate spatiotemporal optical vortices^30–32^ and applied in new types of optical tweezers^33^ and quantum optical telecommunications^34^.

## Theoretical model

We collided a relativistic plasma flying mirror with an oblique laser beam, considering laser beams carrying both SAM and OAM, to observe the change in AM before and after reflection.

## PIC simulation of linearly polarized LG mode

**Figure 1:**
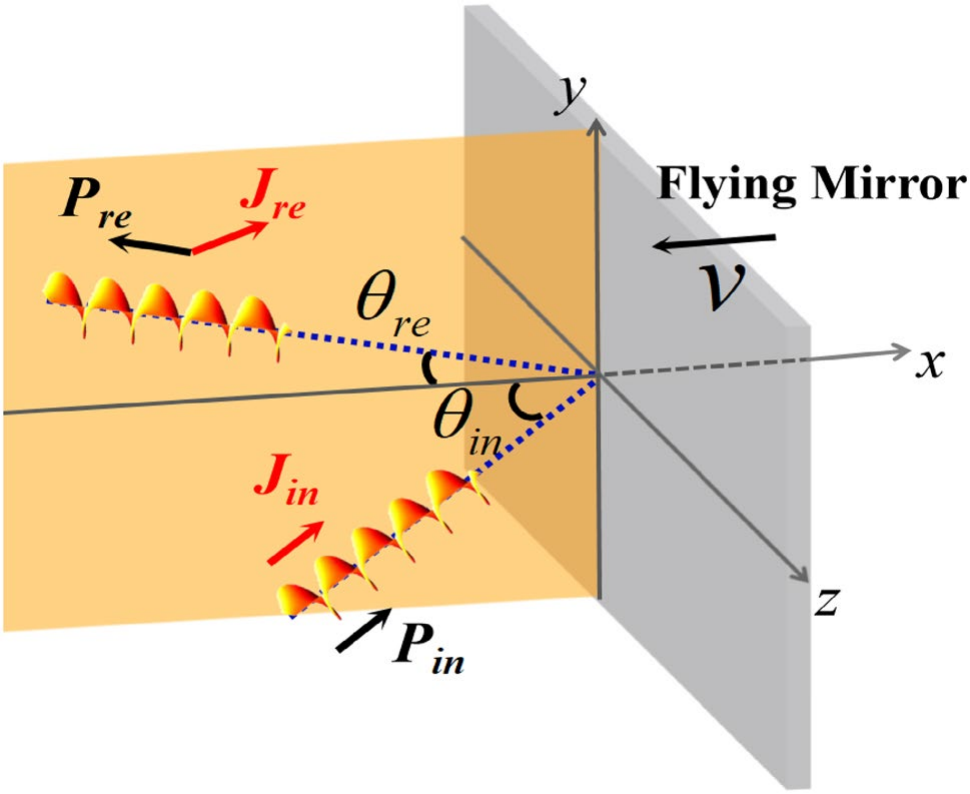
Schematic of the proposed mechanism. A vortex laser beam (with LM **P**_**in**_ and nonzero AM **J**_**in**_) is obliquely incident at an angle ${\theta }_{\mathrm{in}}$ and reflected (with LM **P**_**re**_ and AM **J**_**re**_) by the flying mirror (the gray part moves in the − *x* direction). The angle of reflection ${\theta }_{re}$ is significantly less than ${\theta }_{in}$. Before the laser collides with the plasma mirror, **P**_**in**_ and **J**_**in**_ are collinear. After the reflection from the flying mirror, **P**_**re**_ and **J**_**re**_ are noncollinear.

The reflection process was investigated using the three-dimensional (3D) particle-in-cell (PIC) code EPOCH^35^. The well-known Laguerre–Gaussian (LG_01_) mode is used as a typical vortex beam, and it can be expressed by^36,37^

$$u\left(L{G}_{01}\right)=\sqrt{\frac{2}{\pi }}\frac{r\sqrt{2}}{{w}^{2}\left(x\right)}\mathrm{exp}\left[-\frac{{r}^{2}}{{w}^{2}\left(x\right)}\right]\mathrm{exp}\left(i\varphi \right)\mathrm{exp}\left[\frac{i{k}_{0}{r}^{2}x}{2\left({x}^{2}+{x}_{r}^{2}\right)}\right]\mathit{exp}\left[-2i{\mathrm{tan}}^{-1}\left(\frac{x}{{x}_{r}}\right)\right]{\mathrm{sin}}^{2}\left(\frac{\pi t}{2\tau }\right)$$

when it propagates in the *x* direction. Here, $r=\sqrt{{y}^{2}+{z}^{2}}$; $\varphi =\mathrm{arctan}(z/y)$; ${k}_{0}=2\pi /{\lambda }_{0}$ is the wave number; and the wavelength of the incident beam is ${\lambda }_{0}=0.8 \mathrm{\mu m}$. Moreover, $w\left(x\right)={w}_{0}\sqrt{{(x}^{2}+{x}_{r}^{2})/{x}_{r}^{2}}$,$\mathrm{where } {w}_{0}=6.25{\lambda }_{0}$ is the beam waist radius and ${x}_{r}$ is the Rayleigh length. The longitudinal profile was composed of ${\mathrm{sin}}^{2}(\pi t/2\tau )$ and $\tau = 26.7\mathrm{ fs}$. The dimensionless amplitude ${a}_{0}=e{E}_{0}/{m}_{e}c{\omega }_{0}=1$, where $e$, ${m}_{e}$, ${\omega }_{0}$, ${E}_{0}$, and $c$ are the electron charge, electron mass, laser frequency, peak amplitude of the laser field, and light velocity in vacuum, respectively. In our PIC simulation, a linearly polarized LG_01_ beam obliquely impinged on the flying mirror with ${\theta }_{in}=\pi /6$ (relative to the *x* axis), as shown in Fig. 1. Initially, the LG laser was injected from $x=-5{\lambda }_{0}$, and the flying mirror was upright and occupied the domain in $13.75{\lambda }_{0}$< *x* < $15{\lambda }_{0}$, $-18.75{\lambda }_{0}$< *y* < $18.75{\lambda }_{0}$, $-10{\lambda }_{0}$< *z* < $10{\lambda }_{0}$. The electron density of the mirror was 20 ${n}_{c}$ to ensure that the laser pulse was reflected completely^17,38^, here, ${n}_{c}$ is the critical density. The flying mirror moved in the –*x* direction at a speed of $v=0.5\mathrm{c}$. Here, we note that the velocity of the flying mirror was set to 0.5c instead of a more relativistic velocity to save computational resources, that is, to achieve quick separation of the laser from the target and avoid the influence of numerical heating. The simulation box was a $25{\lambda }_{0}$×$50{\lambda }_{0}$ ×$25{\lambda }_{0}$ cuboid corresponding to a window of 400 × 800 × 400 cells in the *x* × *y* × *z* direction, and each cell had 10 macroparticles.

**Figure 2:**
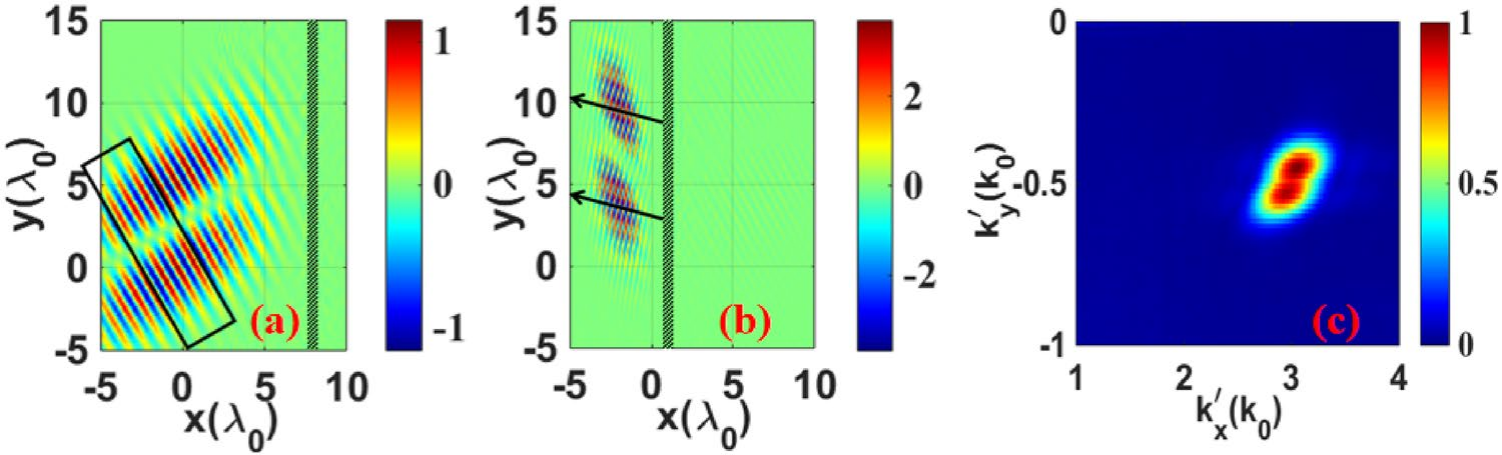
(**a**) Distribution of the incident electric field ${E}_{y}$ in the *x*–*y* plane. The black box shows the electric field selected to calculate the OAM. (**b**) Distribution of the reflected electric field ${E}_{y}^{\mathrm{^{\prime}}}$. The arrow indicates the direction of the LM (the propagation direction of the reflected beam). The length is normalized to ${\lambda }_{0}.$ The field is normalized to ${m}_{e}{\omega }_{0}c/e(4\times 1{0}^{12}\mathrm{V}/\mathrm{m for }{\lambda }_{0}=0.8\mathrm{ \mu m}$). (**c**) The *k*-space spectrum profile of the reflected beam. ${{\varvec{k}}}_{{\varvec{x}}}^{\mathrm{^{\prime}}}\mathrm{ and }{{\varvec{k}}}_{{\varvec{y}}}^{\mathrm{^{\prime}}}$ are the components of the wave vector in the *x* and *y* direction, respectively, normalized to ${k}_{0}$. The intensity is normalized to the peak intensity.

The simulation configurations of the incident and reflected electric fields are shown in Fig. 2a,b, respectively. Compared with the field before reflection, the distribution of the reflected field changed significantly, including the profile and propagation direction. In Fig. 2b, the arrow indicates the direction of the LM of the reflected beam, defined based on the direction of the LM, which must be perpendicular to that of the electromagnetic field. The wavelength of the reflected beam was smaller than that of the incident beam, and the angle of reflection was smaller than the angle of incidence owing to the relativistic effect. As shown in Fig. 2c, the laser was tightly focused, and the longitudinal component of the electromagnetic field could not be ignored; thus, the *k*-space spectrum profile of the reflected field experienced spectral broadening. According to the coordinates of the center of gravity of the laser, the wavelength and emission angle of the reflected beam were ${\lambda }_{1}=0.283 \mathrm{\mu m}$ and ${\theta }_{re}=0.174$ rad, respectively.

Furthermore, ${\lambda }_{1}$ and ${\theta }_{re}$ can be derived from the Doppler shift formulae. Using Lorentz transformation, in the flying mirror frame, the angle of incidence ${\mathrm{tan}\theta }_{\mathrm{in}}^{^{\prime}}=\mathrm{sin}{\theta }_{\mathrm{in}}/(\gamma \mathrm{cos}{\theta }_{\mathrm{in}}+\gamma \beta )$ and the wavelength of the incident beam ${\lambda }_{0}^{^{\prime}}={\lambda }_{0}(1/\left(\gamma +\gamma \beta \mathrm{cos}{\theta }_{\mathrm{in}}\right)$) with the relativistic factor $\gamma =1/\sqrt{1-{\beta }^{2}}$, $\beta =v/c$. In the same frame, the angle of reflection is equal to the incident angle. By inverse Lorentz transformation, in the laboratory frame, the angle of reflection $\mathrm{tan}{\theta }_{\mathrm{re}}={\mathrm{sin}\theta }_{\mathrm{in}}^{^{\prime}}/(\gamma {\mathrm{cos}\theta }_{\mathrm{in}}^{^{\prime}}+\gamma \beta )$, and the wavelength of the reflected beam ${\lambda }_{1}={\lambda }_{0}^{^{\prime}}(1/\left(\gamma +\gamma \beta {\mathrm{cos}\theta }_{\mathrm{in}}^{^{\prime}}\right)$). For ${\theta }_{in}=\pi /6$ and $\beta =0.5, {\theta }_{\text{re}}=0.176$ rad and ${\lambda }_{1}=0.281 {\upmu}{\text{m}}$ were obtained, which are in good agreement with the simulation results.

**Figure 3:**
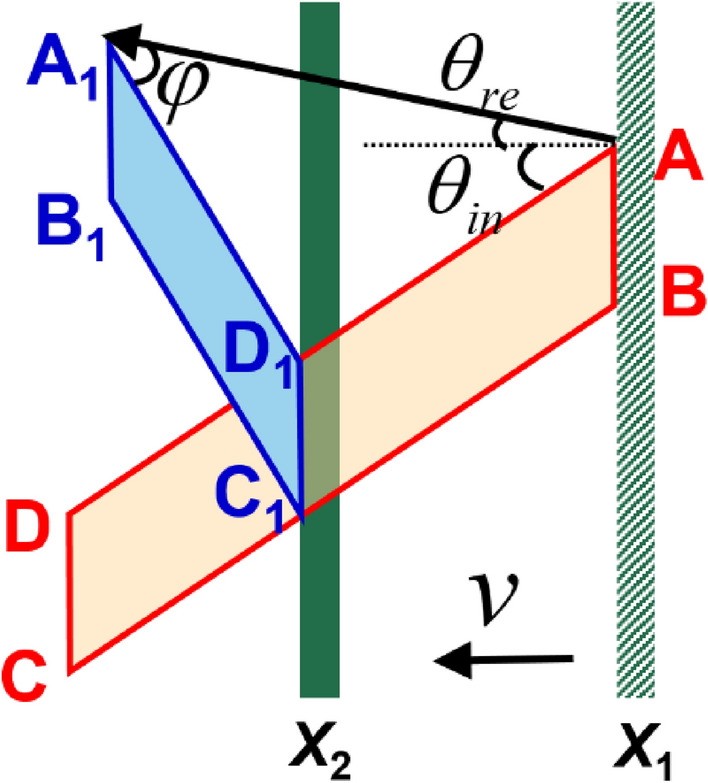
Schematic of the profile change of the electric field. The red quadrilateral (ABCD) represents the profile of the incident electric field; the direction of the vector $\overrightarrow{\mathrm{DA}}$ is defined as the incident profile direction. The blue quadrilateral (A_1_B_1_C_1_D_1_) represents the profile of the reflected electric field and the direction of the vector $\overrightarrow{{\mathrm{D}}_{1}{\mathrm{A}}_{1}}$ is defined as the reflection profile direction. The angle between the LM and the profile of the electric field is defined as $\varphi$, where $s\mathrm{in}\varphi /\mathrm{sin}(\varphi +{\theta }_{in}+{\theta }_{re})=\beta \mathrm{cos}{\theta }_{in}/\mathrm{cos}{\theta }_{re}$. X_1_ represents the position of the flying mirror at the beginning of the interaction between the vortex beam and the flying mirror, and X_2_ represents the position of the flying mirror after the interaction.

Given the angle of reflection, the changes in the beam profile can be well explained. As shown in Fig. 3, the red quadrilateral (ABCD) was chosen from Fig. 2a. The incident beam was reflected along $\overrightarrow{{\mathrm{AA}}_{1}}$ (the direction of the LM), and the angle between the LM and profile of the electric field was defined as $\varphi$, where $\mathrm{sin}\varphi /\mathrm{sin}(\varphi +{\theta }_{in}+{\theta }_{re})=\beta \mathrm{cos}{\theta }_{in}/\mathrm{cos}{\theta }_{re}$. When plane AB began to interact with the flying mirror, the target was at *X*_1_ and $\varphi =0$. When the flying mirror was moved to *X*_2_, the beam was completely reflected. At this moment, planes AB and CD were transformed to A_1_B_1_ and C_1_D_1_, respectively. Therefore, the profile of the reflected field became the blue quadrilateral A_1_B_1_C_1_D_1_. According to this analysis, the angle between the LM and the profile of the reflected field should be $\varphi =0.403$ rad, which is in good agreement with the simulation result of $\varphi =0.424$ rad, as shown in Fig. 2b.

The aforementioned results confirm that the emission angle, wavelength, and distribution of the reflected beam observed in the simulation agree with the theoretical expectations. In the following section, we focus on AM and examine the time history of the average OAM per photon of the electromagnetic field.

**Figure 4:**
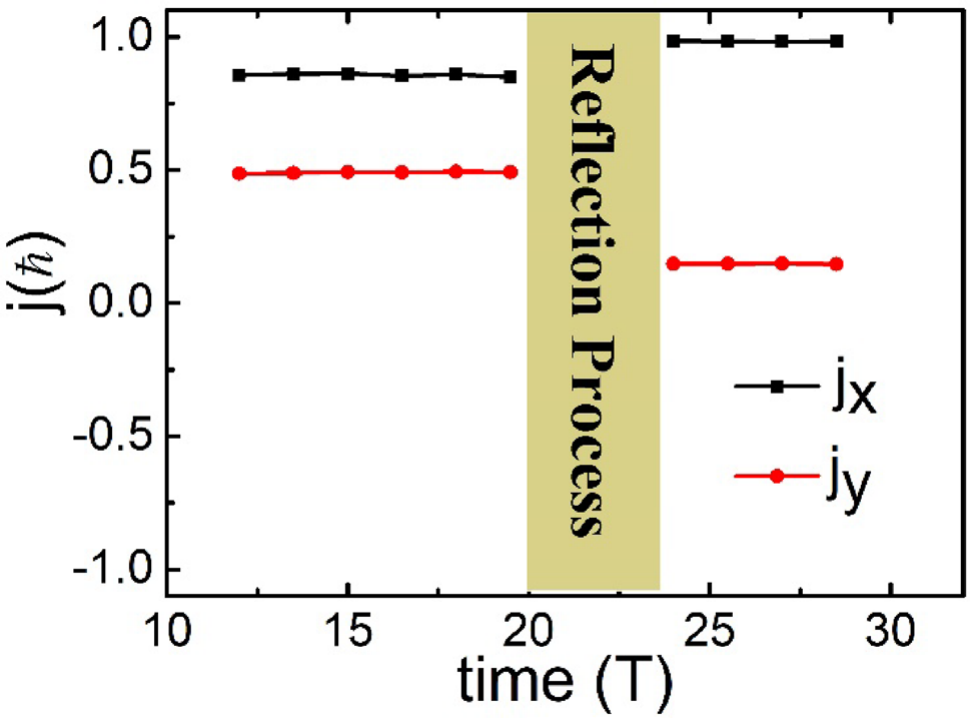
Time history of the average OAM carried by a photon. The black and red dotted lines are the OAM in the *x* and *y* directions, respectively. Time is normalized to the period (T) of the laser beam and the OAM is normalized to $\mathrm{\hslash }$.

The average AM carried by a photon was calculated based on the field data from the PIC simulations using $j={\varepsilon }_{0}{\int }_{V}r\times (E\times B)\mathrm{dV}/\mathrm{N}$, where ${\varepsilon }_{0}$ is the vacuum dielectric permittivity; *r* is the position vector; *E* and *B* are the electric and magnetic fields, respectively; V refers to the entire simulation region for integration; and $\mathrm{N}$ is the number of photons of the entire laser beam. To accurately calculate the AM, we selected the electromagnetic fields during three periods in the middle part of the laser beam, as shown in Fig. 2a. To display the simulation results intuitively, the angles investigated in this study were all angles along the + *x* axis. Figure 4 shows the time history of the OAM carried by a photon; ${j}_{x}$ and ${j}_{y}$ refer to the two components of the OAM in the *x* and *y* directions, respectively. At $t=12 T$, the selected part of the vortex beam was incident on the left boundary of the simulation box. Before the vortex beam interacted with the flying mirror, ${j}_{x}=0.857$ and ${j}_{y}=0.491$ were constant from $t=12 T$ to $t=19.5 T$. Therefore, the direction of the OAM of the incident beam was 0.520 rad, which was collinear with the LM (${\theta }_{in}=\pi /6$ rad) of the incident beam. At $t=24 T$, the selected laser was completely reflected (the same three periods as the incident beam); ${j}_{x}=0.983$ and ${j}_{y}=0.147$ remained constant from $t=24 T$ to $t=30 T$. Therefore, the direction of the OAM of the reflected beam was 0.148 rad, thus indicating noncollinearity with the LM of the reflected beam (according to the aforementioned result of $2.968$ rad). The angle between the LM and OAM is ${\Theta }_{0.5c}=0.320 \mathrm{rad}.$ Essentially, in addition to the longitudinal AM in the propagation direction, transverse AM in the perpendicular propagation direction was generated by the interaction between the vortex laser and the flying mirror. This noncollinear phenomenon results from the profile change of the laser electromagnetic field caused by the double Doppler shift.

## Verification and conclusion

To further verify the aforementioned conclusions, we also considered the cases of CP-Gaussian (carrying only SAM) and CP-LG lasers (carrying both SAM and OAM) for comparison. Circular polarization includes left- and right-handed polarizations, as noted by the LCP and RCP. In the simulations, except for the polarization state, the other parameters were consistent with those in the aforementioned LP-LG case. The results for the LM and AM vectors of the LP-LG, CP-Gaussian, and CP-LG modes are listed in Table 1. The results show that the LM of the reflected beam was always the same as that of the previous simulation, and the angle of reflection for all cases was consistent with the theoretical expectation (${\theta }_{\text{re}}=0.176$ rad); however, the direction of the AM was different for different cases. In the case of the LCP Gaussian mode, the SAM carried by the reflected beam was collinear with the LM but in the opposite direction. In the case of the RCP-Gaussian mode, the SAM carried by the reflected beam was collinear with the LM and in the same direction. The SAM was always collinear with the LM of the laser in this process, which verifies that SAM is an intrinsic characteristic of the CP-Gaussian laser beam. In the case of the $l=1$ LCP-LG mode, before reflection, the average SAM carried by a photon was in the same direction and numerically equal to the average OAM carried by a photon, and the total average AM (2 $\hslash$) carried by a photon of the incident beam was in the same direction as the LM. After reflection, the AM carried by the reflected beam was no longer collinear with the LM. In the case of the $l=1$ RCP-LG mode, before reflection, the average SAM carried by a photon was in the opposite direction and numerically equal to the average OAM carried by a photon; therefore, the laser beam did not carry AM. After reflection, because the OAM carried by the reflected beam was no longer collinear with the LM, the average AM carried by a photon was nonzero and almost perpendicular to the LM of the reflected beam. It should be noted that, as shown in Table 1, the cases of the CP-LG mode seemed to satisfy the conservation of AM. In fact, the total AM after reflection satisfies the vector superposition rule of SAM and OAM. That is, the total AM originated from the SAM in the case of the CP-Gaussian and from the OAM in the case of the LP-LG, which further verifies that the OAM is non-collinear with the LM.

**Table 1:**
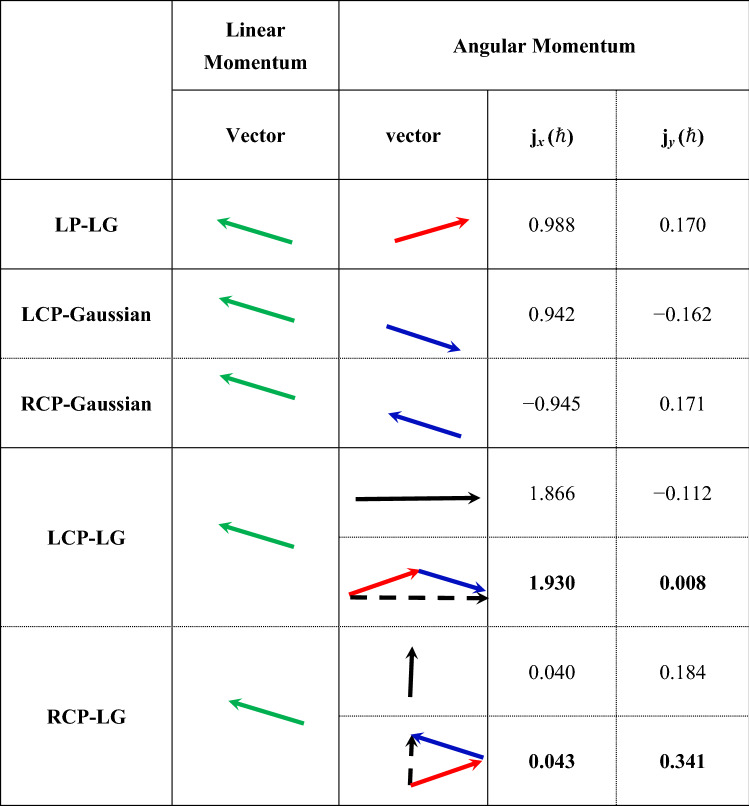
Linear and angular momentums of the reflected beam for different modes of the incident beam.

The green arrows represent the LM directions. The red arrow represents the OAM in the linearly polarized LG (LP-LG) mode. The blue arrows represent SAM in circularly polarized Gaussian (CP-Gaussian) modes. The solid black arrows represent the AM in the case of circularly polarized LG (CP-LG) modes, and the dotted arrows (data in bold are superimposed) represent the results of the vector superposition of OAM in the LP-LG case and SAM in the CP-Gaussian case. Circular polarization includes left- and right-handed polarizations, as noted by the LCP and RCP.

Compared with the static reflecting plane, wherein AM and LM are always collinear, the result of the interaction between vortex beams and the flying mirror shows that the noncollinearity of the AM and LM is positively related to the incident angle and velocity of the flying mirror. When the incidence angle of the laser beam was constant, the high velocity of the flying mirror contributed to the angle between the OAM and the LM. When the velocity of the flying mirror was constant, as the incident angle increased, the laser beam experienced a greater transverse shear force, thereby resulting in a much larger angle between the OAM and LM.

We studied the reflection process of a vortex beam from a flying mirror based on a 3D PIC simulation. After collision with the flying mirror, the SAM carried by the reflected beam was always collinear with the LM, and we confirmed that the SAM carried by the laser beam was related only to its polarization mode. However, as the laser was squeezed, the distribution of the electromagnetic field changed and the OAM became noncollinear with the LM. Recently, spatiotemporal optical vortices have attracted considerable attention owing to their unique properties, such as carrying transverse AM. The reflected beam carrying transverse AM is a new physical phenomenon that is expected to be used to generate ultra-short and ultra-strong spatiotemporal optical vortices, and provides a new degree of freedom to explore the physical effects of the OAM. Furthermore, the incident angle of the laser and the velocity of the plasma mirror can be set to arbitrary values to generate a laser beam with an OAM at any angle to the LM.

## Data Availability

All data generated or analyzed during this study are included in this published article.
